# Identification of Radial Glia Progenitors in the Developing and Adult Retina of Sharks

**DOI:** 10.3389/fnana.2016.00065

**Published:** 2016-06-20

**Authors:** Nuria Sánchez-Farías, Eva Candal

**Affiliations:** Grupo BRAINSHARK, Departamento de Bioloxía Celular e Ecoloxía, Universidade de Santiago de CompostelaSantiago de Compostela, Spain

**Keywords:** Müller glia, fishes, adult neurogenesis, development, GFAP, DCX

## Abstract

Neural stem cells give rise to transient progenitors termed neuroepithelial cells (NECs) and radial glial cells (RGCs). RGCs represent the major source of neurons, glia and adult stem cells in several regions of the central nervous system (CNS). RGCs are mostly transient in mammals, but they are widely maintained in the adult CNS of fishes, where they continue to be morphologically similar to RGCs in the mammalian brain and fulfill similar roles as progenitors and guide for migrating neurons. The retina of fishes offers an exceptional model to approach the study of adult neurogenesis because of the presence of constitutive proliferation from the ciliary marginal zone (CMZ), containing NECs, and from adult glial cells with radial morphology (the Müller glia). However, the cellular hierarchies and precise contribution of different types of progenitors to adult neurogenesis remain unsolved. We have analyzed the transition from NECs to RGCs and RGC differentiation in the retina of the cartilaginous fish *Scyliorhinus canicula*, which offers a particularly good spatial and temporal frame to investigate this process. We have characterized progenitor and adult RGCs by immunohistochemical detection of glial markers as glial fibrillary acidic protein (GFAP) and glutamine synthetase (GS). We have compared the emergence and localization of glial markers with that of proliferating cell nuclear antigen (PCNA, a proliferation maker) and Doublecortin (DCX, which increases at early stages of neuronal differentiation). During retinal development, GFAP-immunoreactive NECs located in the most peripheral CMZ (CMZp) codistribute with DCX-immunonegative cells. GFAP-immunoreactive RGCs and Müller cells are located in successive more central parts of the retina and codistribute with DCX- and DCX/GS-immunoreactive cells, respectively. The same types of progenitors are found in juveniles, suggesting that the contribution of the CMZ to adult neurogenesis implies a transition through the radial glia (RG) state.

## Introduction

In the developing brain of mammals, neural stem cells give rise to progenitors termed neuroepithelial cells (NECs), which in their turn produce radial glia (RG) progenitors. The finding that radial glial cells (RGCs) serve as progenitor cells in development (Malatesta et al., 2000) changed radically the prevailing notion about their function. While these cells had been first considered as guiding cables for migrating neurons towards their final destinations, they are now accepted to be the major source of all the main lineages (neurons, astrocytes, oligodendrocytes, ependymocytes and even adult neural stem cells) in the central nervous system (CNS; see Hartfuss et al., 2001; Doetsch, 2003; Malatesta et al., 2008; Malatesta and Götz, 2013) both under physiological and regenerative conditions. Importantly, this fact has led to the misperception that all RGCs are stem cells, even if only a minority of them self-renew an indefinite number of times and persist for the entire life of the organism (reviewed in Götz et al., 2015). Therefore, the cellular hierarchies involving different types of progenitors and the precise contribution of the RG to adult neurogenesis has been matter of intense research (Kriegstein and Götz, 2003; Ever and Gaiano, 2005; Pilz et al., 2013; Borrell and Götz, 2014; Paridaen and Huttner, 2014; De Juan Romero and Borrell, 2015; Ninkovic and Götz, 2015).

The retina has been increasingly used to approach this question because of the presence of adult constitutive and/or regenerative neurogenesis from different supplies depending on the vertebrate group: the retinal pigmented epithelium, the ciliary marginal zone (CMZ) and a single type of glial cell with radial morphology, the Müller glia (reviewed in Karl and Reh, 2010; Wohl et al., 2012; Than-Trong and Bally-Cuif, 2015; Todd et al., 2016).

The CMZ has been defined in birds, reptiles, amphibians and fishes as a circumference of progenitor cells that persist until the adulthood, located at the edge of the retina between the ciliary epithelium (CE) and the neural retina. It has been suggested that multipotency of CMZ cells diminished through the course of evolution from fish to mammals (Kubota et al., 2002). In fishes, the CMZ generates all retinal cell subtypes, including the Müller glia (Raymond et al., 2006). The spatio-temporal pattern of expression of various stem cell and progenitor markers within the CMZ of zebrafish (Raymond et al., 2006) revealed that the CMZ is arranged in concentric rings of gradually increased commitment, i.e., neural stem cells are located in the most peripheral zone, nearby the CE, while progressively more fate-restricted progenitors are successively located toward the central (differentiated) retina. This spatially ordered arrangement of neural stem cells, NECs and increasingly committed cells could help to follow the progeny of individual progenitor cells from the embryo into postnatal life, which has been technically challenging in the brain of mammals due to the long migration that separates the final progeny from progenitors (e.g., interneurons of the olfactory bulb from progenitors in the walls of the lateral ventricles).

On their hand, Müller glial cells have processes that span the entire thickness of all retinal layers, supporting significantly the retinal function and maintaining visual attainment in the growing eye (Mack et al., 1998; Bringmann et al., 2006; Jadhav et al., 2009; Gallina et al., 2014). Müller glial cells have been identified as constitutive progenitors in the normal retina of fish and as a source of retinal regeneration in fish, chicks and rodents when stimulated by retinal damage or growth factors (Wohl et al., 2012; Gallina et al., 2014; Todd et al., 2016). Müller cells have a limited potential to regenerate neurons in the avian and mammalian retina, which contrast with the significant neurogenic capacity of Müller glia in the fish retina (Gallina et al., 2014). In fishes, Müller cells participate in sustained constitutive proliferation in the adult to generate a population of rod photoreceptor progenitors that give rise to rods throughout the entire life of the fish (Bernardos et al., 2007; Stenkamp, 2011). Müller cells are mostly quiescent, but after injury, they re-enter the cell cycle (Raymond et al., 2006), de-differentiate, and can serve as multipotent retinal stem cells that generate not only photoreceptor progenitors but also all other retinal cell types *in vivo* (for review, see Wu et al., 2001; Fischer and Reh, 2003; Raymond et al., 2006; Bernardos et al., 2007; Wohl et al., 2012; Lenkowski and Raymond, 2014). The fact that, in constitutive neurogenesis, adult Müller cells give rise only to photoreceptors prompted the hypothesis that CMZ cells must generate other types of neurons without a transition through the RG state.

In this context, a deep characterization of progenitor heterogeneity within the CMZ and the identification of molecular changes at the NEC to RGC transition and the subsequent RGC differentiation during development should help to determine if this process also occurs in the mature retina, i.e., whether RG progenitors contribute or not to adult neurogenesis. The retina of the elasmobranch fish *Scyliorhinus canicula* offers an exceptional model to investigate this questions due to several reasons: (1) as in all vertebrates, retinogenesis involves the generation of a layered structure where different types of neurons and a single type of glia become highly organized; (2) as in other fishes, proliferation becomes restricted to the CMZ, where continuous proliferation permits the successive addition of concentric rings of new cells, including neurons and Müller cells; (3) as in other fishes, the predominant glial cell type in the healthy retina is the Müller glia, which shows morphological characteristics of RGCs; and (4) in contrast to fast developing teleosts, it presents a protracted period of proliferation in the central retina, and a long-lasting transition zone (TZ) bordering the CMZ that contain both proliferating and early differentiated cells (Ferreiro-Galve et al., 2010; Sánchez-Farías and Candal, 2015).

We have characterized progenitor and adult RGCs by immunohistochemical detection of glial markers as the glial fibrillary acidic protein (GFAP) and glutamine synthetase (GS). GFAP, an intermediate filament (IF) protein typically found in mature astrocytes, has been increasingly used to label RG progenitors within the CNS (Middeldorp and Hol, 2011). Several studies have described the expression of GFAP in RG progenitors during development of the CNS of some species (reviewed in Kriegstein and Alvarez-Buylla, 2009; Than-Trong and Bally-Cuif, 2015), and also within neurogenic niches of the adult mammalian brain such as the subventricular zone of the lateral ventricles of the telencephalon (Doetsch et al., 1999; Imura et al., 2003; Garcia et al., 2004; Kriegstein and Alvarez-Buylla, 2009) and the subgranular zone of the hippocampus (Seri et al., 2001; Steiner et al., 2006). The presence of GFAP has been additionally proved through *in vivo* assays in progenitors that give rise to proliferating neural precursors in the postnatal olfactory bulb, hippocampus, and cerebral cortex (Ganat et al., 2006). GFAP has been also observed in Nestin-expressing progenitors in neurospheres derived from the adult rat CE (Das et al., 2006). In the retina, most descriptions of GFAP expression have been focused on Müller glia identification in adult stages in mammals (Kumpulainen et al., 1983; Björklund and Dahl, 1985; Schnitzer, 1985; Sarthy et al., 1991), reptiles (Todd et al., 2016) and fishes (Linser et al., 1985), where it was described in young and mature Müller glia, but not in NECs in the CMZ or in de-differentiated Müller glia after retinal damage (for a review, see Than-Trong and Bally-Cuif, 2015). However, only a few studies have addressed GFAP expression during retinal development or throughout lifespan in mammals (Ling and Stone, 1988; Sarthy et al., 1991) and zebrafish (Bernardos and Raymond, 2006; Arenzana et al., 2011). On its hand, GS has been extensively reported in young and mature Müller cells (but not in early RG progenitors) in both the developing and adult retina of zebrafish (Mack et al., 1998; Peterson et al., 2001; Thummel et al., 2008), and *S. canicula* (Bejarano-Escobar et al., 2012). We additionally explored the emergence and localization of GFAP immunoreactivity with respect to that of the proliferating cell nuclear antigen (PCNA, a proliferation marker) and Doublecortin (DCX), an early marker of differentiation of specific cells in the retina of sharks (see Sánchez-Farías and Candal, 2015).

## Materials and Methods

### Experimental Animals

Embryos, juveniles, and one adult specimen of *S. canicula* were provided by the *Aquarium Finisterrae* in A Coruña, the Acuario de O Grove in Pontevedra and the Acuario de Gijón (Spain). Additionally, embryos of *S. canicula* were supplied by the Station Biologique de Roscoff (France). The following embryos were used: stage 22 (1), 24 (1), 25 (1), stage 26 (1), stage 27 (4), stage 28 (1), stage 29 (3), stage 30 (6), stage 31 (3), stage 32 (4), and prehatching (PH) (4). Moreover, 3 juveniles (about 10.7 cm) and one adult (about 33 cm in total length) were also used. Eggs from different broods, juveniles, and the adult were raised in fresh seawater tanks in standard conditions of temperature (10–18°C), pH (7.5–8.5), salinity (35 g/L) and 12:12 h day/night cycle. Embryos were identified by their external features using a stereoscopic microscope, following the descriptions in Ballard et al. (1993). For more information about the correspondence between embryonic stages and body size, gestation period and hatching, see Ferreiro-Galve et al. (2010). Adequate measures were taken to minimize animal pain or discomfort. All procedures conformed to the guidelines established by the European Communities Council Directive of 22 September 2010 (2010/63/UE) and by the Spanish Royal Decree 53/2013 for animal experimentation, and were approved by the Ethics Committee of the University of Santiago de Compostela.

### Tissue Preparation

Embryos up to stage 32 were anesthetized with 0.5% tricainemetanesulfonate (MS-222; Sigma, St. Louis, MO, USA) in seawater and separated from the yolk before fixation in 4% paraformaldehyde (PFA) in elasmobranch’s phosphate buffer [EPB: 0.1 M phosphate buffer (PB) containing 1.75% urea, pH 7.4] for 24–48 h, depending on the stage of development. Embryos from stage 33 onwards, juveniles, and the adult were deeply anesthetized with MSS-222 and then perfused intracardially with elasmobranch’s Ringer solution (see Ferreiro-Galve et al., 2008) followed by PFA 4% in EPB. The eyes of PH (stages 33 and 34) embryos, juveniles, and the adult were removed and postfixed in PFA 4%. After that, the eyes were rinsed in phosphate buffer saline (PBS), cryoprotected with 30% sucrose in PB, embedded in NEG 50^TM^ (Thermo Scientific, Kalamazoo, MI, USA), frozen with liquid nitrogen-cooled isopentane and cut on a cryostat. Parallel series of transverse and sagittal sections (16–18 μm thick) were mounted on Superfrost Plus slides (Menzel-Glässer^®^, Madison, WI, USA).

### Immunofluorescence

For heat induced epitope retrieval, sections were pre-treated with 0.01 M citrate buffer pH 6.0 for 30 min at 95°C, and allowed to cool for 20–30 min at room temperature (RT). Sections were rinsed twice in 0.05 M Tris-buffered saline (TBS) pH 7.4 for 5 min each, and incubated overnight at RT with the following primary antibodies (see Table 1): rabbit polyclonal anti-GFAP, mouse monoclonal anti-GS, rabbit polyclonal anti-DCX, goat polyclonal anti-DCX, and mouse monoclonal anti-PCNA. Then, sections were rinsed twice in 0.05 M TBS pH 7.4, and incubated in the appropriate fluorescent dye-labeled secondary antibody (see Table 2) for 1 h, at RT. Sections were rinsed in TBS for 30 min and in distillated water (twice for 5 min), they were allowed to dry for 2 h at 37°C, and mounted in MOWIOL^®^ 4–88 Reagent (Calbiochem, MerkKGaA, Darmstadt, Germany). All dilutions were made with TBS containing 15% normal donkey serum, 0.2% Triton X-100 (Sigma) and 4% bovine serum albumin (BSA, Sigma). All incubations were carried out in a humid chamber. Double immunofluorescence with primary antibodies raised in the same specie was performed as described in Tornehave et al. (2000). No immunostaining was detected when the primary or secondary antiserum were omitted.

**Table 1:** **Primary antibodies**.

Primary antibody	Source	Working dilution
GFAP	Polyclonal rabbit anti-GFAP	1500
	DakoCytomation
	Catalog number: Z 0334, Lot: 00082268
GS	Monoclonal mouse anti-GS	1:100
	Merk-Millipore
	Catalog number: MAB302, Lot: 2090111
DCX	Polyclonal rabbit anti-DCX	1:300
	Cell Signaling Technology
	Catalog number: 4604S, Lot: 3
DCX	Polyclonal goat anti-DCX	1:100
	Santa Cruz Biotechnology
	Catalog number: sc-8066, Lot: C2513
PCNA	Monoclonal mouse anti-PCNA	1:800
	Sigma-Aldrich
	Catalog number: P8825, Lot: 082M4844

**Table 2:** **Secondary antibodies**.

Primary antibody	Source	Working dilution
546-conjugated donkey	Molecular probes	1:100
anti-rabbit (DAR^546^)	Catalog number: A10040
546-conjugated donkey	Molecular probes	1:100
anti-mouse (DAM^546^)	Catalog number: A10036
488-conjugated donkey	Molecular probes	1:100
anti-rabbit (DAR^488^)	Catalog number: A21206
488-conjugated donkey	Molecular probes	1:100
anti-goat (DAG^488^)	Catalog number: A11055
488-conjugated donkey	Molecular probes	1:100
anti-mouse (DAM^488^)	Catalog number: A21202
633-conjugated donkey	Molecular probes	1:100
anti-mouse (DAM^633^)	Catalog number: A31571

### Specificity of the Antibodies

The polyclonal anti-GFAP antibody is a purified immunoglobulin fraction of rabbit antiserum generated to bovine spinal cord GFAP. This anti-GFAP antibody has been previously used in *S. canicula* as a glial marker (Sueiro et al., 2007; Quintana-Urzainqui et al., 2014).

The two different anti-DCX polyclonal antibodies used here have been previously used in the retina of *S. canicula* (for more details, see Sánchez-Farías and Candal, 2015).

The monoclonal anti-PCNA antibody (Sigma) recognizes a protein of 36 KDa corresponding to the acidic non-histone auxiliary protein of DNA polymerase, according to the manufacturer. It is expressed in the nuclei of interphase (G1-S-G2) cells (reviewed in Candal et al., 2005). The anti-PCNA antibody was previously used to label proliferating cells in the retina in *S. canicula* (Ferreiro-Galve et al., 2010; Sánchez-Farías and Candal, 2015).

The monoclonal anti-GS antibody (Merk-Millipore) is a purified immunoglobulin fraction of 45 kDa from sheep brain generated in mouse. This antibody cross-reacts for many species including human, mouse, rat and sheep (manufacturer’s information). In *S. canicula* this antibody has been previously found in Müller glial cells (Bejarano-Escobar et al., 2012).

### Image Acquisition and Analysis

Labeled fluorescent sections were studied with the spectral confocal laser scanning microscopes TCS-SP2 and SP5 (Leica, Wetzlar, Germany). Some sections were photographed with an epifluorescence photomicroscope Olympus AX70 fitted with an Olympus DP70 color digital camera. Photographs were minimally adjusted for brightness and contrast by using Adobe Photoshop CS5 Software (Adobe, San Jose, CA, USA).

## Results

We have characterized RGCs in the retina of *S. canicula* in the frame of three developmental periods previously described in this species (see Ferreiro-Galve et al., 2010), which have served as a useful context for comparison with retinal development in other vertebrates.

### Embryos up to Stage 29

**Figure 1:**
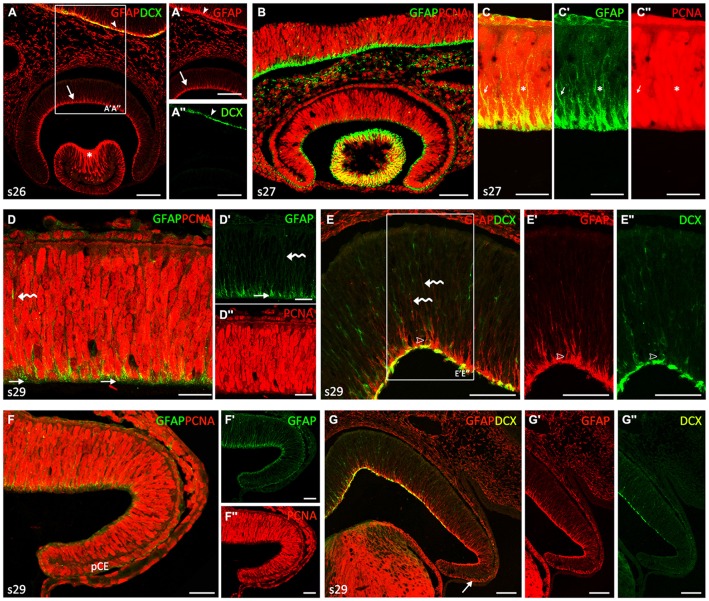
**Comparison of glial fibrillary acidic protein (GFAP), proliferating cell nuclear antigen (PCNA) and Doublecortin (DCX) immunoreactivities in neuroepithelial non-layered retina from stages 26–29. (A–C″)** Vertical (transverse) sections along the dorsoventral axis of the retina of stage 26–27 embryos. **(A–A″)** Vertical section of the central retina showing GFAP immunoreactivity at stage 26 (arrow in **A,A′**), in DCX-negative cells. Arrowheads indicate GFAP and DCX immunoreactivity in the encephalon. Asterisk indicates strong GFAP-imunoreactivity in lens fibers. **(B)** Vertical section of the retina at stage 27 showing that most Neuroepithelial cells (NECs) are proliferating cells (PCNA-ir). Note that GFAP immunoreactivity is less intense than that observed in the brain. **(C–C″)** Detail of the retina to show that GFAP and PCNA do not co-localize within the cell. Arrows and asterisks indicate double labeled cells. **(D–G″)** Vertical sections along the dorsoventral axis of a stage 29 retina. **(D–D″)** Double GFAP/PCNA immunolabeling in sections of the central retina reveals GFAP-ir pyramidal endfeet (arrows in **D,D′**) that codistribute with weakly PCNA-ir cells **(D″)**. Curved arrows indicate isolated faintly GFAP-ir processes. **(E–E″)** Section to show that some DCX-ir cells appear to be associated with GFAP-ir endfeet (open arrowheads).Curved arrows indicate isolated faintly GFAP-ir processes. GFAP and DCX are observed in the emergent optic fiber layer (OFL), albeit they do not co-localize. **(F–F″)** In the peripheral retina the pattern of these markers is similar to that found in the central retina at previous developmental stages, i.e., GFAP immunoreactivity is less intense and restricted to the endfeet. GFAP immunoreactivity is present in cells of the presumptive ciliary epithelium (pCE). **(G–G″)** DXC immunoreactivity is absent in the most peripheral retina. Arrow indicates GFAP immunoreactivity in the pCE. Scale bars: 25 μm in **(D–D″, F–F″)**; 50 μm in **(C, E–E″)**; 75 μm in **(A–B, G–G″)**.

During the *first developmental* period, which includes embryos up to stage 29 (Figure 1), the retina showed a neuroepithelial pseudostratified appearance, where cells were arranged in radial columns. Cells immunoreactive (-ir) to GFAP were recognized in the neuroepithelial retina. From stage 22–26, weak GFAP immunoreactivity was restricted to the innermost third of most cell processes, which ended in the innermost part of the neuroepithelium, i.e., in the basal (vitreal) surface (arrows in Figures 1A,A′). Note that GFAP-immunoreactivity in retinal NECs was slightly less intense to that observed in other encephalic regions at the same developmental period (arrowheads in Figures 1A,A′). GFAP-ir cells were also found in lens fibers (asterisk in Figure 1A). At stage 26, cells in the retina were immunonegative to DCX, although DCX immunoreactivity was clearly identified in other encephalic regions (arrowheads in Figures 1A,A″). First DCX-ir cells were observed from stage 27 onwards (not shown). At this stage, interphase nuclei (PCNA-ir) were located at different positions giving the neuroepithelium a stratified aspect (Figures 1B,C) and mitosis were only found in the outer (ventricular) margin (see Sánchez-Farías and Candal, 2015), which matches the definition of interkinetic nuclear migration (IKNM; see “Discussion” Section). Though GFAP immunoreactivity was observed in proliferating (PCNA-ir) cells, colocalization was not observed in the same cell structures since PCNA is a nuclear marker and GFAP is a cytoskeletal protein located in the cytoplasm (arrows in Figures 1C–C″).

At the end of this period (stage 29) the thickness of the still proliferating neuroepithelium had increased (Figures 1D–G″). Although the retina did not show any apparent regional differentiation or layering (i.e., terminal cell differentiation had not started and different cell layers were therefore not organized), several spatial differences in the distribution of GFAP-ir, PCNA-ir and DCX-ir cells were noted at this stage. In the central part of the retina, weakly-GFAP immunoreactivity was observed in some isolated processes in the neuroblastic layer extending toward the outer retina (curved arrows in Figures 1D,D′,E). GFAP-ir pyramidal endfeet were mainly aligned in the innermost part of the retina (arrows in Figures 1D,D′), where the intensity of PCNA immunoreactivity decreased in most cells (Figure 1D″). Some cells at this location were also DCX-ir and appeared to be associated to GFAP-ir endfeet (open arrowheads in Figures 1E–E″). Immunoreactivity for both GFAP and DCX was observed in the emergent optic fiber layer (OFL) though co-localization between these markers was not evident.

In the most peripheral retina (Figures 1F–G″), the immunoreactivity pattern of these markers was similar to that observed in central regions at the beginning of this period, i.e., GFAP immunoreactivity was less intense and restricted to cells endfeet (Figures 1F–G′), PCNA immunoreactivity was widely found in interphase cells (Figures 1F,F″) and DCX immunoreactivity was absent (Figures 1G,G″; see also Sánchez-Farías and Candal, 2015). Interestingly, faint GFAP immunoreactivity was also observed in cells of the presumptive CE, especially in their basal domain (Figure 1F; arrow in Figure 1G).

### Stages 30–32

**Figure 2:**
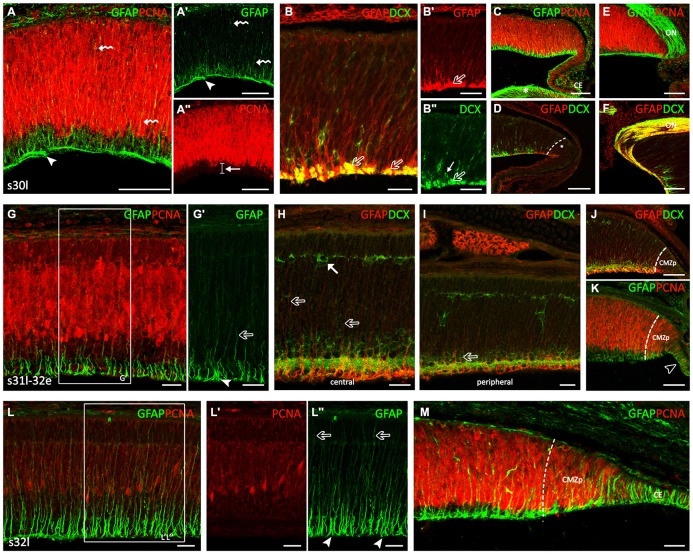
**GFAP, PCNA and DCX immunoreactivity patterns in the retina of *S. canicula* during the layering process.** Vertical (transversal) sections along the dorsoventral axis of the retina. **(A–F)** Vertical sections of the retina at stage 30. **(A–A″)** Intense GFAP immunoreactivity is observed in some processes in the middle and outer neuroblastic regions (curved arrows in **A,A′**). GFAP-ir cell processes are observed in the inner third region, where pale PCNA-ir nuclei are placed (arrow in **A″**). Arrowheads in **(A,A′)** point to GFAP-ir cell processes in the OFL, where DCX-ir axons from rounded-ganglion cells are observed. **(B–B″)** Detail of the central retina to show DCX and GFAP immunoeactivities in the OFL (open arrows). Arrow in **(B″)** indicates a DCX-ir amacrine cell. **(C,D)** Details of the peripheral retina, where the GFAP immunoreactivity is reminiscent of that found in the central retina at previous stages. Strong GFAP immunoreactivity is found in the lens (asterisk in **C**) and in the CE **(C)**. Asterisk in **(D)** indicates de CMZ in a peripheral region (CMZp). **(E,F)** GFAP-immunoreactivity in the optic nerve (ON) head. **(G–K)** Vertical sections of the retina at stage late 31-early 32 (31l-32e). **(G,G′)** Detail of the central retina to show that GFAP immunoreactivity increases along cell processes (open arrows) and in endfeet (arrowhead). **(H)** Detail of the central retina. Some GFAP-ir-apical directed processes (open arrows) reach an emerging horizontal cell layer (HCL) occupied by DCX-ir cells (arrow). **(I)** In peripheral regions the portion of GFAP-ir apical-directed processes is shorter (open arrow). **(J)** The CMZp is occupied by strong GFAP immunoreactivity. **(K)** GFAP immunoreactivity is observed in the CE (open arrowhead). **(L–M)** Vertical sections of the retina at stage 32l. **(L–L″)** GFAP immunoreactivity is found in the inner half of cell processes and in the better defined endfeet of Müller cells, close to the vitreal region (arrowheads in **L″**). GFAP immunoreactivity increases in the apical-directed processes and some of them reach the apical surface (open arrows in **L″**). **(M)** In the peripheral retina, GFAP and DCX immunoreactivity patterns are similar to those found at previous stages in the central retina. Note the high intensity of GFAP-ir cells in the CE. Scale bars: 25 μm in **(B,B″,G–I,L–M)**; 75 μm in **(A–A″,B,C–F,J,K)**.

The *second developmental* period (Figure 2) is defined by the progressive layering of the central part of the retina, which in turn allows clearly defining the extension of the CMZ (non-layered) and the TZ, which separates the CMZ from the central (layered) retina. At stage 30, the central part of the inner retina becomes layered by the gradual formation of the inner plexiform layer (IPL), which separates the ganglion cell layer (GCL) from the inner part of the inner nuclear layer (INLi). The distribution of GFAP immunoreactivity was similar to that observed earlier at stage 29 (Figures 2A,A′). Intense GFAP immunoreactivity was observed in some processes in the middle and outer neuroblastic region (curved arrows in Figures 2A,A′) and in cells processes at the inner third region of the central retina, which roughly coincided with the area where PCNA-immunonegative nuclei were located (arrow in Figure 2A″). Intense GFAP immunoreactivity was also observed in thick cell endfeet and cell processes located in the OFL (arrowheads in Figures 2A,A′), where DCX-ir axons from ganglion cells were also observed (open arrows in Figures 2B,B″). The distribution of GFAP in the peripheral retina resembled that found at previous stages (Figures 2C,D; compare with Figures 1F,G). Very strong GFAP-immunoreactivity was also found in the lens (asterisk in Figure 2C), in the CE (Figure 2C) and in the optic nerve (ON) head (Figures 2E,F). These processes were organized over the surface of DCX-ir ganglion cell axon bundles as they coursed into the ON head (Figure 2F). GFAP was also observed in the ON at later developmental stages. The distribution of DCX in the peripheral retina allowed us to subdivide the CMZ in a peripheral region (peripheral CMZ, CMZp) containing DCX-negative cells (Figure 2D) and an adjacent middle region (middle CMZ, CMZm) where DCX immunoreactivity resembles that described for the central retina of stage 29 embryos.

By late stage 31, the appearance of GFAP immunoreactivity in the central retina changed considerably (Figures 2G–H). While in previous developmental stages GFAP-ir filaments were disheveled, at this stage GFAP immunoreactivity increased and was neatly arranged along cell processes and endfeet. Again, the strongest GFAP immunoreactivity was restricted to the inner third (arrowhead in Figure 2G′), while less intense GFAP immunoreactivity was found in processes extending through the IPL towards the outer retina (open arrow in Figure 2G′). Of note, GFAP-ir processes were absent from the OFL (which eased identifying GFAP immunoreactivity in distinct endfeet). In the central retina, some GFAP-ir apical-directed processes (open arrows in Figure 2H) reach an emerging horizontal cell layer (HCL) occupied by DCX-ir cells (arrow in Figure 2H), while in peripheral regions the portion of apical processes showing GFAP immunoreactivity was always shorter (about one-third of the cell; open arrows in Figure 2I). Intense GFAP-ir and DCX-ir outward-directed processes were observed in the peripheral retina except in the CMZp, which was occupied by strong GFAP-ir and DCX-immunonegative processes (Figure 2J). As noted above, copious GFAP immunoreactivity was found in cells of the CE (open arrowhead in Figure 2K). Interestingly, PCNA immunoreactivity was more intense in the peripheral retina than in the CE, the latter containing a high number of faintly PCNA-ir cells.

At stage 32 the retinal layering was well-defined at central regions with the presence of a noticeable outer plexiform layer (OPL) and the prospective outer nuclear layer (ONL). PCNA immunoreactivity has considerably decreased (Figures 2L,L′). The retina of these embryos showed GFAP immunoreactivity in the inner half of cell processes and in the now better defined endfeet of Müller cells, close to the vitreal region (arrowheads in Figure 2L″). GFAP immunoreactivity increased in the apical-directed processes and some GFAP-ir processes reached the apical surface (open arrows in Figure 2L″). In the peripheral retina (Figure 2M), the morphology of GFAP-ir cells resembled that observed in the central retina at previous developmental stages and the CE showed a strong GFAP immunoreactivity.

### Stages 33–34

**Figure 3:**
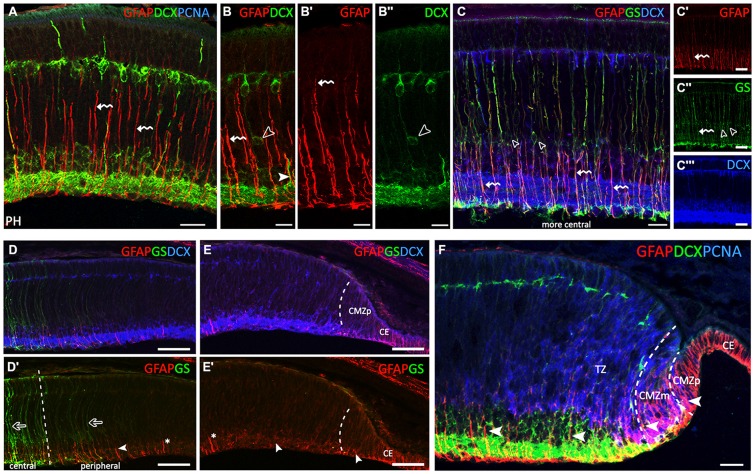
**GFAP, glutamine synthetase (GS), PCNA and DCX immunoreactivity patterns in the retina of *S. canicula* at prehatching (PH) stages. (A–F)** Vertical sections along the dorsoventral axis of the retina of PH embryos. **(A)** Strong GFAP immunoreactivity in radially oriented Müller cell processes (curved arrows) and in their endfeet. **(B–B″)** Curved arrows indicate radially oriented GFAP-ir Müller cell processes. DCX-ir cells are placed at intermediate positions associated with GFAP-ir radial Müller cell processes (arrowheads in **B,B″**). DCX-ir cells are located in the central retina in PCNA-negative areas, in the ganglion cell layer (GCL), inner part of the INL (INLi), outer part of the INL (INLo) and HCL. **(C–C′″)** In the most central part of the retina, GS-ir Müller cell bodies are placed in the INLi (open arrowheads in **C,C″**). Müller cell somas are GFAP- and DCX-immunonegative (**C,C′″**, respectively). Radial Müller cell processes are GS- and GFAP-ir (curved arrows in **C–C″**). **(D,D′)** GFAP-ir processes gradually decrease in length from the central to the peripheral retina (arrowhead). GS immunoreactivity gradually decreases towards the periphery (open arrows). **(E,E′)** GFAP-immunoreactivity is restricted to the inner part of cell processes (arrowheads). No GS-ir cells or processes are observed in the most peripheral region. For reference, asterisk in **(D′,E′)** indicates the same cell process. **(F)** The layered part of the transition zone (TZ) includes PCNA-negative and DCX-ir cells. Intense GFAP-ir processes are observed in the endfeet in the OFL, in the CMZp and in the CE. Arrowheads show changes in GFAP immunoreactivity in cells in the peripheral retina (CMZp, CMZm and TZ). Scale bars: 25 μm in **(A–C′″,F)**; 50 μm in **(D–E′)**.

In the course of the *third developmental* period, layering progressed to almost the entire circumference of the retina, which acquired the mature organization typical of postembryonic stages (Figure 3). Since there were no significant differences in GFAP-immunoreactivity between stages 33 and 34, we considered both as PH embryos.

The central retina contained only a few PCNA-ir cells (Figure 3A). In these embryos, GFAP immunoreactivity was restricted to radially oriented Müller cell processes, especially to their two inner thirds (curved arrows in Figures 3A–B′), and also to their endfeet. However, Müller cell bodies, mainly located in the INL, were immunonegative to GFAP. Most DCX-immunoreactivity was found in ganglion, amacrine, bipolar and horizontal cells (see Sánchez-Farías and Candal, 2015), and also in some DCX-ir cell bodies identified at intermediate positions closely associated with GFAP-ir RG processes (open arrowheads in Figures 3B,B″ and arrowhead in Figure 3B, respectively). Note that GFAP-ir processes gradually increased in length from the peripheral to the central retina (compare Figures 3C–E).

With the aim to ascertain if different intensities of GFAP immunoreactivity in cell processes and/or the location of GFAP-ir filaments along cell processes could be related to Müller cell maturation we double-labeled the retina for GFAP and GS which has been previously described in mature Müller cells in the retina of *S. canicula* (Bejarano-Escobar et al., 2012). In the most central retina, GS-ir Müller cell bodies were found in the outer part of the INLi (open arrowheads in Figures 3C,C″). Müller cell bodies were immunonegative to GFAP (Figure 3C′) and DCX (Figure 3C′″). However, radial processes from Müller cells were both GS- and GFAP-ir (curved arrows in Figures 3C–C″), immunoreactivity to GFAP being much more intense in inner cell processes. GS immunoreactivity gradually decreased in peripheral regions (open arrows in Figure 3D′), where intense GFAP immunoreactivity was still present though restricted to the inner third of cell processes and endfeet (arrowhead in Figure 3D′), which resembled the distribution of GFAP in the central retina at earlier stages (compare with Figure 2G). No GS-ir cells or processes were found in the most peripheral region (Figure 3E), where faint GFAP-ir processes appeared disheveled (arrowheads in Figure 3E′; compare with Figure 1D). In the non-layered part of the TZ (Figure 3F), faint GFAP-ir processes were observed co-localizing with PCNA-ir nuclei. In the TZ close to the CMZ, some DCX-ir neuroblasts were also found at intermediate positions (Figure 3F). The inner (layered) part of the TZ was comprised of PCNA-negative and DCX-ir cells in both the GCL and INLi, and DCX-ir processes in the IPL and axons in the OFL. In this region, strongly GFAP-ir processes were observed through the GCL, IPL and INLi, and in endfeet in the OFL. In the CMZm the pattern of GFAP immunoreactivity was similar to that found in the central retina at stage 29. In turn, the CMZp was characterized by GFAP-ir cell processes spanning the non-layered neuroepithelium and the absence of DCX- immunoreactivity.

### Juveniles and Adults

**Figure 4:**
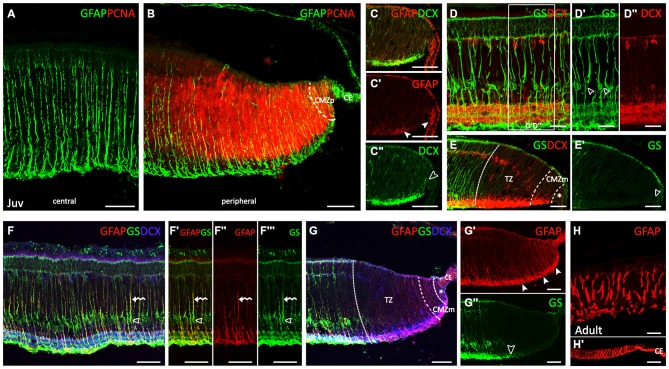
**Comparison of spatiotemporal patterns of GFAP, GS, PCNA and DCX in the juvenile and adult retina of *S. canicula*. (A–G″)** Vertical sections along the dorsoventral axis of the retina of juveniles. **(A)** Very strong GFAP-ir Müller cell processes are observed in the central retina. **(B)** GFAP immunoreactivity is observed in the CMZp, as in PH embryos. **(C–C″)** Details of GFAP and DCX labeling in the ciliary marginal zone (CMZ). Arrowheads in **(C′)** point to GFAP immunoreactivity in the CE and in the CMZ. Open arrowhead in **(C″)** points to intense DCX immunoreactivity bordering the CMZp. **(D–D″)** GS-ir Müller cell bodies in the central retina closely associated with DCX-ir cells. Open arrowheads in D′ point to GS-ir Müller cell bodies. **(E,E′)** GS immunoreactivity is absent in cells and processes in the TZ, CMZm and CMZp (arrowhead). DCX immunoreactivity is absent in the CMZp (asterisk). **(F–F′″)** Detail of the central retina. Open arrowheads indicate GS-ir cell bodies. Curved arrows indicate GS-ir or GFAP-ir cell procesees. **(G–G″)** GFAP and GS immunoreactivities in the peripheral retina are similar to that found in PH embryos. Asterisk in **(G)** indicates de CMZp. Arrowheads in **(G′)** indicate GFAP-ir processes in the peripheral retina. Open arrowhead in **(G″)** indicates the point from which GS immunorativity is no longer detected. **(H,H′)** Vertical sections along the dorsoventral axis of the retina in adults. Strong GFAP-ir Müller cell processes in the central retina **(H)**. GFAP immunoreactivity is also observed in the CMZ and in the CE **(H′)**. Scale bars: 25 μm in **(D–D″,H)**; 50 μm in **(A–C″,E–G″)**; 100 μm in **(H′)**.

Very strong GFAP immunoreactivity was observed in Müller cells in juveniles (Figure 4A). GFAP-ir processes course radially through the entire thickness of the central retina from the inner limiting membrane (ILM) to the outer limiting membrane (OLM). The pattern of GFAP immunoreactivity in the peripheral retina was similar that observed in the same region in PH embryos (Figures 4B,C–C″). Changes in cell morphology and accordingly, GFAP immunoreactivity observed throughout development are recapitulated from the CMZp to the central retina (see Figures 4A,B). The cell bodies of Müller cells in the central retina were GS-ir (Figure 4D, open arrowheads in Figure 4D′). DCX immunoreactivity extended to the retina bordering the CMZp (asterisk in Figure 4E). As in PH embryos, neither GS-ir cell bodies nor processes were found in the TZ (Figures 4E,E′). GS-ir Müller cell bodies were observed in the central retina of juveniles (open arrowheads in Figures 4F–F′″). Co-localization between both glial cell markers, GS and GFAP, was found in the radial processes of Müller cells (curved arrows in Figures 4F–F′″). The distribution of GFAP and GS in the peripheral retina was the same found in the peripheral retina in PH embryos (Figures 4G–G″). Again, the organization of GFAP in the peripheral retina was the same found in the central retina in early stages. In adults, the thickness of the retina has considerably decreased (Figure 4H). GFAP immunoreactivity in the central retina was maintained in Müller cell processes as in juveniles. GFAP immunoreactivity was also observed in the periphery, including the CMZ, and in the CE (Figure 4H′).

## Discussion

During development, neural stem cells gradually change in potential to give rise to progenitors (NECs and RGCs) that in turn generate different types of neurons and glial cells at different times and locations (De Filippis and Binda, 2012; Paridaen and Huttner, 2014; Götz et al., 2015). Both NECs and RGCs (collectively known as apical progenitors) show similar distribution and morphological features in the embryo. However, NECs are pre-neurogenic cells: they have limited self-renewal ability *in vivo* and divide symmetrically to generate more NECs. As the epithelium thickens, NECs suffer a transition to RGCs which divide asymmetrically a limited number of times to generate neurons and glial cells (reviewed in Kriegstein and Alvarez-Buylla, 2009; Paridaen and Huttner, 2014; Götz et al., 2015). However, some differences exist between species and between neurogenic niches regarding the main types of progenitor cells and the progeny they will produce within the nervous system.

We aimed to characterize progenitor heterogeneity within the CMZ and molecular changes at the NEC to RGC transition during development, which should help to determine if this process also occurs in the adult retina. We have previously defined the CMZ by the lack of layering that characterizes the central (differentiated, mature) retina. In this work, the distribution of DCX in the peripheral retina allowed us to additionally subdivide the CMZ in a CMZp containing DCX-immunonegative cells and an adjacent CMZm where DCX immunoreactivity resembles that described for the central retina of stage 29 embryos. These regions could correspond with the peripheral and CMZm defined in zebrafish (Raymond et al., 2006) where cell arrangement reflects the temporal sequence of retinogenesis (i.e., the CMZ-peripheral contains multipotent progenitors while the CMZ-middle contains proliferating but fate-restricted progenitors).

### Neuroepithelial (Pre-neurogenic) Retina

**Figure 5:**
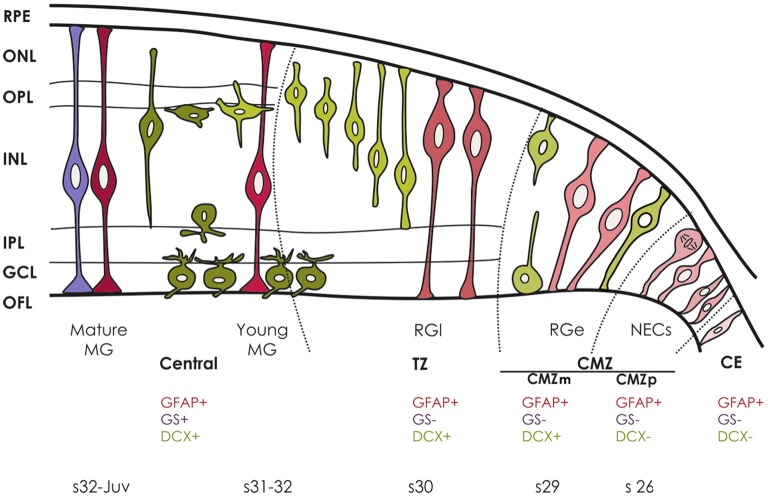
**Sequence of DCX, GFAP and GS immunoreactivity during maturation of the retina in *S. canicula*.** DCX-ir cells are represented in green, GFAP-ir cells are represented in pink and GS-ir cells are represented in purple. Different shades of the same color represent different states of cell maturation.

GFAP immunoreactivity appears very early during retinal development in *S. canicula*. It was first observed in the vitreal endfeet of cells that we interpreted as NECs because of two main reasons. First, during early development all cells were proliferating, with nuclei located at different positions giving the retina a pseudostratified appearance, which matches the definition of IKNM characteristic of NECs. Indeed, IKNM has been previously reported in NECs in an extensive variety of regions in the CNS (Baye and Link, 2007; Del Bene, 2011; Kosodo, 2012; Reiner et al., 2012; Spear and Erickson, 2012). Second, the neurogenic process has not begun since DCX (a marker of the neuronal lineage; see Sánchez-Farías and Candal, 2015) is not detected until stage 27. The neuroepithelial appearance of the retina at early stages was very similar to that observed in other pseudostratified epitheliums along the CNS before the neurogenesis process begins (Götz and Huttner, 2005; Paridaen and Huttner, 2014). A similar pattern was observed in the CMZp of the mature retina (see Figure 5), which therefore appears to contain pre-neurogenic multipotent progenitors.

In mammals GFAP is not expressed in embryonic retinal cells (Sarthy et al., 1991), while in zebrafish GFAP expressing cells (followed by GFAP-driven transgenes; Bernardos and Raymond, 2006) were described in the retina as early as 24 h post fertilization, that in zebrafish precedes the onset of histological differentiation. However, if these cells correspond to NEC (pre-neurogenic) or RG (neurogenic) progenitors has not been determined. GFAP expression was not analyzed in the developing retina of other vertebrate groups (see “Introduction” Section), which preclude further comparison.

### Early RGCs in the Retina

At stage 29 the first signs of regional differentiation can be observed in the basis of PCNA and DCX immunoreactivity (see Figures 1D,E), which indicates that neurogenesis has commenced. The same pattern is observed in the CMZm in the mature retina (Figure 5). According with previous works concerning embryonic neurogenesis (Alvarez-Buylla et al., 2001; Götz and Huttner, 2005; Kriegstein and Alvarez-Buylla, 2009; Sild and Ruthazer, 2011), NECs elongate and transform into RGCs when progenitors change from a symmetric to an asymmetric mode of cell division that coincides with the beginning of neurogenesis (Kriegstein and Götz, 2003; Rakic, 2003; Paridaen and Huttner, 2014). Therefore, pale GFAP-ir processes of RGCs co-distributing with strongly DCX-ir cells observed at this stage could correspond to *early RGCs* according with the nomenclature used in the cortex by Kriegstein and Alvarez-Buylla (2009). GFAP has been already described in RGCs in the ventricular zone at the cerebral cortex during development (nicely reviewed in Kriegstein and Alvarez-Buylla, 2009), but to our knowledge, it has not been described in early RGCs in the developing retina before.

### Transition from RG to Müller Glia During Retinal Layering Process

At stage 30, differentiation begins in the innermost part of the central retina which was occupied by PCNA-negative cells, some of them being also DCX-ir. GFAP immunoreactivity was more intense in the inner third of cells processes in the area occupied by PCNA-negative nuclei (Figure 2). This pattern was also observed in the TZ in the mature retina (see Figure 5). Because of the increase in GFAP expression in this region and the disorganized aspect of GFAP-ir filaments, we will refer these cells as *late RGCs*.

Between stages 31 and 32, some significant differences in the appearance of GFAP immunoreactivity in cell processes take place, so that GFAP-ir filaments are now neatly arranged in their thick basal-directed apical processes. These cells thus acquire the typical morphology characteristic of *young Müller cells* coinciding with the maturation of the central retina (see Figure 5): ganglion and amacrine cells differentiate and are clearly separated in their respective layers by the presence of a conspicuous IPL. Our results would be in line with the ultrastructural study in the brown banded bamboo shark, where Müller cells have been also identified at early stages (Harahush et al., 2009) simultaneously with ganglion cell differentiation. Our results are also compatible with previously reported roles of Müller cells in the histological organization of the developing retina, in the correct establishment of the neuronal circuits and as a scaffold for young migrating neurons (Willbold et al., 1997; Bringmann et al., 2006). Indeed, we also observed some radial DCX-ir bipolar cell processes closely associated with these GFAP-ir glial cell processes.

Of note, Bejarano-Escobar et al. (2012) did not observed the typical morphology of Müller cells in the retina of *S. canicula* before the stage late 32 by using GS immunohistochemistry. However, these could correspond to *mature Müller cells* in the basis of observations in the retina of other species. Indeed, while structural analyses suggest that Müller cells appear early in development of the retina of zebrafish, birth-dating analyses and the appearance of biochemical markers of maturation of various glial markers including GS, suggest that mature Müller cells appear late in development, after the retina is morphologically established (Peterson et al., 2001).

At stage 32, the central retina reaches its mature organization. GFAP immunoreactivity was intense in the inner two thirds of Müller cells, while GS-ir processes in the same cells spanned throughout the entire retina (Figures 3, 5). Both GFAP and GS immunoreactivity were maintained in these cells from this stage until the adulthood. While only weakly GFAP-ir Müller processes have been observed in the mature retina of the stingray (Linser et al., 1985), the presence of GFAP immunoreactivity in radial processes of Müller cells has been found in the mature retina of a few teleosts such as the goldfish (Bignami, 1984; Nona et al., 1989), zebrafish (Yazulla and Studholme, 2001; Bernardos and Raymond, 2006; Arenzana et al., 2011), and pipefish (Linser et al., 1985). Since the retina in fishes grows during the entire life of the animal, the presence of GFAP-ir in these cells and their capacity to proliferate and act as late retinal progenitor cells has led to the proposal that Müller cells in the adult retina can serve the same role as postnatal RG (late progenitor cells) found in neurogenic niches in the adult CNS of mammals. In fishes, Müller cells give rise to late-stage retinal progenitors of the rod photoreceptor lineage. In *S. canicula*, PCNA has been found in cell nuclei in the central mature retina, in a position that could correspond to progenitors of the photoreceptor lineage (Ferreiro-Galve et al., 2010). Müller cells that contribute to adult neurogenesis have been also reported in mammals. In the adult mammalian retina, where constitutive proliferation is limited, GFAP was found in astrocytes but Müller cells do not express GFAP or contain only low amounts of this protein (Sarthy et al., 1991). While GFAP immunoreactivity was very weak or even non-existent (Björklund and Dahl, 1985; Sarthy et al., 1991) and GFAP has been classically used as a tool to differentiate astrocytes (GFAP+) from Müller cells (GFAP−) (Lemmon and Rieser, 1983; Gariano et al., 1996), following injury or in response to the loss of retinal neurons, Müller cells become reactive, showing an upregulation in the expression of GFAP (Eisenfeld et al., 1984; Björklund and Dahl, 1985; Erickson et al., 1987; Bringmann et al., 2009; Bargagna-Mohan et al., 2010). GFAP has been also reported in the Müller glia in reptiles, but their possible role as progenitor cells have not been reported so far in this group.

### Progenitor Cells in the CMZ and Ciliary Epithelium of the Mature Retina

Though Müller cells can be neurogenic under certain conditions, they are not considered the main progenitor cells for retina in the adult because of their late appearance during the neurogenic period. The other neurogenic niche in the retina of fishes, amphibians and turtles is the CMZ, which persists in the adulthood and contain progenitor cells that proliferate and add new cells to the peripheral retina (Perron and Harris, 2000; Fischer et al., 2013; Centanin and Wittbrodt, 2014; Than-Trong and Bally-Cuif, 2015; Todd et al., 2016). A CMZ that persists into the adulthood was also found in the avian retina (Kubota et al., 2002; Fischer and Reh, 2003; Fischer and Bongini, 2010) though the neurogenic capacity of its progenitors is limited. In mammals, non-pigmented cells at the peripheral edge of the retina have been considered to serve as a CMZ-like zone, since they show stem cell characteristics when they are cultured *in vitro*. However, it has been reported that they do not represent a significant source of regeneration (Ahmad et al., 2000; Perron and Harris, 2000; Tropepe et al., 2000; Kubota et al., 2002; Fischer and Reh, 2003; Fischer and Bongini, 2010; Todd et al., 2016).

In *S. canicula*, different types of GFAP-immunoreactive progenitors have been identified in the CMZ, based on their differential morphology, location and expression of DCX. Cells lacking DCX in the CMZp are interpreted as pre-neurogenic and then should correspond with NECs. In teleost fishes, neuroepithelial progenitors in the CMZ express both neural stem cells and progenitor markers (Nestin, BLBP, Sox2) but not GFAP. However, other glial markers (vimentin) have been recently reported in the marginal region of the neural retina of the adult human (Bhatia et al., 2009).

In the teleost fish medaka, tracing individual CMZ cells from embryos and juveniles demonstrated that CMZ contains multipotent retinal stem cells that generate all the neuronal cell types and Müller glia, which in turn can act as neural progenitors (Centanin et al., 2011; Centanin and Wittbrodt, 2014). This result together with the fact that Müller glial cells become rapidly quiescent after they are generated from the CMZ and after constitutive activation, they give rise only to photoreceptors, has led to the hypothesis that the chief contribution of the CMZ (non-glial) cells to generate all types of adult-born neurons does not implies an obligatory transition through the RG state. In *S. canicula* GFAP and DCX immunoreactivity was found in cells with radial morphology bordering the CMZp. These cells can represent progenitor RG, which suggest that CMZ contribution to adult neurogenesis involves a transition through RG progenitors.

In medaka, the pool of stem cells is maintained through a predominantly asymmetric mode of cell division, though it has been suggested that stem cells also divide symmetrically to expand the number of active stem cells during eye growth (Centanin et al., 2014). If these features apply to the CMZ of other vertebrate groups (with different competence for adult neurogenesis), or if an additionally source of stem cells exist to maintain or increase the pool of stem cells remains to be addressed (Than-Trong and Bally-Cuif, 2015). In *S. canicula*, most cells in the CMZ are strongly PCNA-ir, a feature that does not match with the long-lasting maintenance of stem cells. Interestingly, cells of the CE show the same phenotypic properties than CMZp cells (Figure 5) except for the fact that scarce PCNA-ir cells were observed in this structure, which raises the hypothesis that some cells in the CE could serve as a quiescent source of stem cells for retinal development and growth.

## Conclusions and Future Directions

We showed here that differences in the intensity and appearance of GFAP immunoreactivity, together with the combined distribution of GFAP with that of GS, DCX and PCNA within the spatio-temporal frame provided by the peripheral retina could help to differentiate different types of progenitors in the retina of sharks. NECs are found in the CMZp. They present GFAP immunoreactivity in their endfeet, codistribute with DCX-immunonegative cells and share phenotypic properties with NECs elsewhere in the CNS. Early and late RG are located in the CMZm and TZ, respectively, co-distributing with strongly DCX-immunoreactive cells. They are characterized by the presence of disheveled GFAP immunoreactivity in endfeet and in the inner third of cell processes extending towards the outer retina. Early and late Müller cells are found in progressively more central regions of the mature (layered) retina. These cells are characterized by the presence of neatly arranged GFAP immunoreactivity in cell processes and GS-immunoreactivity in somas and cell processes. The same types of progenitors are found in juvenile specimens, suggesting that the contribution of the CMZ cells to generate all types of adult-born neurons implies a transition through the RG state even in adults. On the other hand, the presence of GFAP in cells of the CE suggests that these cells could contribute to the continuous renewal of the pool of stem cells present in the CMZ. The detailed neurochemical characterization of different progenitor cells within each region of the peripheral retina of sharks could help to characterize progenitor cells in other regions of the CNS, where the absence of a spatio-temporal frame makes difficult to differentiate the transition from one type of progenitor to another.

## Author Contributions

NSF and EC designed the study; NSF performed the experiments; NSF and EC analyzed the data and wrote the manuscript.

## Conflict of Interest Statement

The authors declare that the research was conducted in the absence of any commercial or financial relationships that could be construed as a potential conflict of interest.

## Abbreviations

CE: ciliary epithelium
CMZ: ciliary marginal zone
CMZm: middle CMZ
CMZp: peripheral CMZ
CNS: central nervous system
DCX: doublecortin
GCL: ganglion cell layer
GFAP: glial fibrillary acidic protein
GS: glutamine synthetase
HCL: horizontal cell layer
IF: intermediate filament
IKNM: interkinetic nuclear migration
ILM: inner limiting membrane
INLi: inner part of the INL
INLo: outer part of the INL
IPL: inner plexiform layer
-ir: immunoreactive
NEC: neuroepithelial cell
OFL: optic fiber layer
OLM: outer limiting membrane
ON: optic nerve
ONL: outer nuclear layer
OPL: outer plexiform layer
pCE: presumptive ciliary epithelium
PCNA: proliferating cell nuclear antigen
PH: prehatching
RG: radial glia
RGC: radial glial cell
TZ: transition zone.
